# Diagnosis despite clinical ambiguity: physicians’ perspectives on the rise in Autism Spectrum disorder incidence

**DOI:** 10.1186/s12888-021-03151-z

**Published:** 2021-03-12

**Authors:** Michael Davidovitch, Dorit Shmueli, Ran Shmuel Rotem, Aviva Mimouni Bloch

**Affiliations:** 1 Child Development, Medical Division and Research Institute, Maccabi Healthcare Services, 27 Hamered St., 6812509 Tel Aviv, Israel; 2 Kahn-Sagol-Maccabi Research and Innovation Institute, Maccabi Healthcare Services, 4 Koifmann St., 6801296 Tel Aviv, Israel; 3 Clalit Child Development Center, Clalit Healthcare Services, 75 Betlehem Rd., 9362410 Jerusalem, Israel; 4 Department of Environmental Health, Harvard T H Chan School of Public Health, Boston, Massachusetts USA; 5 Pediatric Neurology and Developmental Unit, Loewenstein Rehabilitation Hospital, 278 Ahuza St., 4310000 Raanana, Israel; 6 Sackler Faculty of Medicine, Tel Aviv University, Tel Aviv, Israel

**Keywords:** Autism Spectrum disorder, Diagnosis, Physicians’ opinion

## Abstract

**Background:**

To provide insight on physicians’ perspectives concerning recent changes in the incidence and diagnostic process of Autism Spectrum Disorder (ASD) compared to other mental and neurodevelopmental disorders.

**Method:**

A questionnaire was sent to 191 specialists in child neurology and child development, and 200 child psychiatrists in Israel. Information was collected on professional background, as well as on physicians’ opinions concerning the accuracy and rate of ASD diagnosis compared to that of cerebral palsy (CP), mental illness, and Attention Deficit Hyperactivity Disorder (ADHD). For each closed-ended question, a global chi-square test for categorical variables was performed.

**Results:**

115 (60.2%) of specialists in child neurology and development, and 59 (29.5%) of child psychiatrists responded. Most physicians (67.2%) indicated that there was a moderate/significant increase in the incidence of ASD, which was higher than similar responses provided for CP (2.9%, *p* < 0.01) and mental illnesses (14.4%, *p* < 0.01), and similar to responses provided for ADHD (70.1%, *p* = 0.56). 52.8% of physicians believed that in more than 10% of clinical assessments, an ASD diagnosis was given despite an inconclusive evaluation (CP: 8.6%, *p* < 0.01; mental illnesses: 25.8%, *p* = 0.03; ADHD: 68.4%, *p* = 0.03).

**Conclusion:**

The clinicians perceive both ASD and ADHD as over-diagnosed disorders. The shared symptomology between ASD and other disorders, coupled with heightened awareness and public de-stigmatization of ASD and with the availability of ASD-specific services that are not accessible to children diagnosed with other conditions, might lead clinicians to over-diagnose ASD. It is advisable to adopt an approach in which eligibility for treatments is conditional on function, rather than solely on a diagnosis. The medical community should strive for accurate diagnoses and a continuous review of diagnostic criteria.

## Background

Autism Spectrum Disorder (ASD) is a severe disorder with pervasive difficulties in social interaction, restricted communication, and repetitive patterns of behavior [1], resulting in significant personal, familial, and societal impacts. ASD prevalence has increased dramatically in the last three decades. In the U.S., the prevalence at 8 years of age increased from 0.2% during the nineties [2] to recent estimates of 1.85% [3]. Reports from Israel show a similar trend, with cumulative incidence estimates of 1.3% in 2018 [4]. This steady rise in ASD is uncommon for severe developmental disorders. While a genetic basis for the disorder is well documented with hundreds of genes identified as potential etiologic contributors [5], estimates suggest that inheritable and de novo gene variations only account for 10–20% of ASD cases [6]. Recent research has also associated other factors with ASD risk, including advanced parental age, birth complications, maternal pregnancy-related factors, and environmental influences [7]. However, while the cumulative effect of the above factors likely contributes to the rising trend in ASD incidence, ASD has no biological markers, and the diagnosis relies solely on the evaluation of developmental history, questionnaires, clinical observations, and semi-structured tests. This has led some researchers to suggest that there are other, extrinsic determinants related to seeking a diagnosis and to the diagnostic process itself that account for the increasing trend in ASD. These include diagnostic expansion, the inclusion of other neurodevelopmental disorders, diagnostic substitution, diagnostic awareness, diagnostic coupling to services and entitlements, diagnostic resolution, and overdiagnosis [8]. To provide some insight on physicians’ perspectives concerning the possible effects of the above factors, we surveyed specialists in child neurology and development and child psychiatry on recent changes in the incidence and diagnostic process of ASD compared to other mental and neurodevelopmental disorders.

## Methods

A questionnaire was designed to obtain physicians’ opinions on changes in incidence and diagnostic practices for the following conditions: ASD, cerebral palsy (CP), mental illness (anxiety, depression, or schizophrenia), and Attention Deficit Hyperactivity Disorder (ADHD). In the last section, physicians were asked to make recommendations for changes in policy that the Israeli Ministry of Health should implement regarding ASD. Questions are listed in Table 1. The questionnaire was distributed in January and February 2020 to 191 specialists in child neurology and child development, and to 200 child psychiatrists, primarily via email (due to low response rate in the psychiatrists’ group, a paper questionnaire was also distributed to them during a scientific conference). Contact information for the physicians was obtained from the Child Neurology and Development Association and the Child Psychiatry Association, which are both parts of the Israeli Medical Association, and includes most of the specialists in these fields in Israel. The responses were anonymized and returned to the first author of this article.

**Table 1:** Study Questionnaire

	Question	Possible responses					
1	What is your area of specialty	Child neurology and development		Child psychiatry			
2	How many years have you been working in your field?	up to 5		5–10			above 10
3	As part of your work, do you diagnose children with ASD?	Yes		No			
4	Where does the diagnosis process take place?	Public clinic		Private clinic			Both
5	What percentage of your time at work do you spend on ASD diagnosis?	up to 10%		10–50%			above 50%
6	In your opinion, has there been an increase in the number of children diagnosed with ASD?	significant	modest	low	no		do not know
7	Do you think that there is an increase in the percentage of ASD diagnoses given despite the clinical evaluation being inconclusive?	significant	modest	low	no		do not know
8	Of all ASD diagnoses, what percentage of diagnoses do you think were given despite having some doubt?	1–10%	11–20%	21–30%	31–40%	41–50%	Above 50%
9	In cases when an ASD diagnosis is given despite the evaluation being inconclusive, what do you think is the main reason for this?	substitution of diagnosis	to grant autism benefits	diagnostic expansion	familial pressure	diagnostic awareness	other (explain)

Questions 7 and 8 were repeated for cerebral palsy (CP); Attention Deficit Hyperactivity Disorder (ADHD); and anxiety, depression, and schizophrenia (the latter 3 conditions were subsequently grouped to a broader mental illness category);

### ASD diagnosis process

The ASD diagnosis process in Israel must adhere to the following guidelines: 1) the diagnosis should be performed by a pediatric psychiatrist, pediatric neurologist, or a developmental pediatrician, and must include detailed physical, neurological, and developmental evaluations; 2) the ASD diagnosis must be based on the Diagnostic and Statistical Manual of Mental Disorders, 5th Edition (DSM-5) criteria [1]; 3) an independent assessment by a developmental or clinical psychologist must confirm ASD case status; 4) the final report should include a detailed description of the child’s developmental and cognitive status; and 5) inputs from other medical specialists, including speech and language specialists, occupational therapists, and social workers should be included in the evaluation process as needed. The ASD evaluations for children up to the age of 6 are multidisciplinary and take place in child development centers. For older children, the evaluations are usually performed by child psychiatrists or neurologists and child psychologists through the Health Maintenance Organizations, or in hospital-based centers for ASD diagnosis. Some of the assessments take place in private clinics due to long waiting for public services.

#### Statistical analysis

For each closed-ended question, a global chi-square test for categorical variables was performed to determine if statistically significant differences were observed for physicians’ responses across the different neurodevelopmental and mental conditions considered. If statistically significant results were obtained in the global test, post hoc pairwise comparisons using multiple 2 × 2 contingency tables were used to specifically compare physicians’ responses for ASD to each of the other disorders considered.

## Results

Out of 191 experts in child neurology and development and 200 psychiatrists that were contacted, 115 (60.2%) and 59 (29.5%) provided responses, respectively, for a total of 174 physicians (44%) that completed the survey. Most physicians had more than 5 years of practice experience (86% of experts in child neurology and development; 75% of child psychiatrists), overall, 90% of them diagnosed ASD in their practice, and 86% worked in both public and private clinics.

The majority of physicians (67.2%) indicated that there is a moderate or significant increase in the incidence of ASD diagnosis, which was statistically significantly different compared to the number of similar responses provided for CP (2.9%, *p* < 0.01) and other mental disorders (14.4%, p < 0.01), however not compared to ADHD (70.1%, *p* = 0.56) (Table 2). Additionally, more than half of the physicians (52.8%) believed that in more than 10% of clinical evaluations, an ASD diagnosis was given despite the assessments being inconclusive, an estimate which was statistically significantly different than the number of similar responses provided for CP (8.6%, *p* < 0.01) and mental illness (25.8%, *p* = 0.03), as well as ADHD (68.4%, p = 0.03) (Table 3). More than a third of the physicians believed that the reason why clinicians diagnosed ASD in inconclusive cases was because of benefits that children with ASD are entitled to receive (Table 4).

**Table 2:** Physician response to the question of uncertain diagnoses

	Is there an increase in uncertain diagnosis?											
	Significant Increase		Moderate Increase		Sum S + M	Low Increase		No Increase		No answer / Do not know		Sum Low+No+No answer
	ND N (%)	Psy N (%)	ND N (%)	Psy N (%)	ND+ Psy N (%)	ND N (%)	Psy N (%)	ND N (%)	Psy N (%)	ND N (%)	Psy N (%)	ND+ Psy N (%)
ASD	36 (31.3)	21 (35)	38 (33)	22 (36.7)	117 (67.2)	21 (18.3)	6 (10)	13 (18.3)	4 (6.7)	7 (6.1)	6 (10)	57 (32.8)
CP	1 (0.9)	0	3 (2.6)	1 (1.7)	5 (2.9)	15 (13)	2 (3.3)	76 (66.1)	6 10)	20 (17.4)	50 (83.3)	169 (97.1)
Mental Illness	6 (5.2)	1 (1.7)	6 (5.2)	12 (20)	25 (14.4)	22 (19.1)	16 (26.7)	15 (13)	22 (36.7)	66 (57.4)	8 13.3)	149 (85.6)
ADHD	35 (30.4)	21 (35)	47 (40.9)	19 (31.7)	122 (70.1)	20 (17.4)	12 (20)	11 (9.6)	5 (8.3)	2 (1.7)	2 (3.3)	52 (29.9)

*ND* Physicians expert in neurology and development, *Psy* Physicians expert in child psychiatry, *Sum S + M* sum of significant and moderate response, *Sum Low+No+No answer* sum of low, no increase and do not know / no answer, *ASD* autism spectrum disorder, *CP* cerebral palsy, *Mental illness* anxiety or depression or schizophrenia, *ADHD* attention deficit hyperactivity disorder

**Table 3:** Physician response to the question of the percent of uncertain diagnoses

	Percent of Uncertain Diagnosis							
	Up to 10%	11–20%	21–30%	31–40%	41–50%	More than 50%	Sum Above 10%	No answer / Do not know
ASD	25.9	23.5	19.5	2.9	4	2.9	52.8	21.3
CP	37.4	5.7	2.3	0	0.6	0	8.6	54
Mental Illness	23.6	17.8	5.7	1.15	1.15	0	25.8	50.6
ADHD	18.4	31.6	19.5	8.6	3.5	5.2	68.4	13.2

*ASD* autism spectrum disorder, *CP* cerebral palsy, *Mental illness* anxiety or depression or schizophrenia, *ADHD* attention deficit hyperactivity disorder. All *P* values are for comparisons to the number of similar responses provided for the ASD group

**Table 4:** Physicians responses to the reasons for uncertain diagnosis in ASD

Substitution of diagnosis	Autism benefits	Diagnostic expansion	Familial pressure	Diagnostic awareness	Combined reasons	Other reasons	No answer
21 (12.1)	64 (36.8)	37 (21.3)	7 (4)	14 (8)	23 (13.2)	3 (1.7)	5 (2.9)

N (%)

Ninety-two (53%) physicians wrote comments regarding ASD regulations, diagnoses, and services. The main suggestions included: 1. Providing services, treatments, and benefits according to the child’s functional level and for specific deficits in development and not solely based on diagnostic label; 2. Children younger than 3 years of age should receive a temporary diagnosis of ASD with reevaluation at the age of three, since ASD diagnosis may resolve in some children that received ASD diagnosis at a very young age; 3. Permission to diagnose ASD should only be given to highly knowledgeable and experienced psychiatrists, neurologists, psychologists, and other health professionals; 4. Implementation of stricter criteria for ASD diagnosis.

## Discussion

This study describes physicians’ opinions regarding ASD diagnosis in comparison to other behavioral and mental disorders. Most physicians agreed that ASD incidence is increasing, with many attributing some of this increase to diagnoses that were given despite the clinical evaluation being inconclusive. The vast majority of the specialists who answered the survey diagnose ASD in their practice, and indeed when asked about areas with which they were less familiar, most declare that they do not know or did not respond (e.g., psychiatrists answers on cerebral palsy, Table 2).

The risk factors known to be associated with ASD development are genetic variations, perinatal [7] and environmental. Amongst the environmental factors, several toxicants, including endocrine-disrupting chemicals, air pollution, pesticides, and certain pharmaceuticals were reported to impact ASD development [9–12] . Recent reports of screen exposure and less caregiver-child play early in life have been associated with later ASD-like symptoms [13], although the direction and importance of which is unknown.

The overall increase in ASD rates over the last three decades may be the result of several interrelated factors. Awareness of ASD has tremendously increased in recent years amongst medical staff, educators, and parents. In the clinic, parents often express their fear of autism, even though their toddlers might have symptoms of more common conditions, such as expressive language delays. Additionally, symptoms of ASD overlap with those of several other conditions, including intellectual and learning disability, and developmental language disorder [14–16]. Despite the shared symptomology, access to medical and support services for children diagnosed with non-ASD conditions is typically much more limited compared to children diagnosed with ASD. In Israel, children with ASD are eligible for an extended basket of care (14 h weekly) as well as to other treatments and special education services, from their first year of life through the age of 7. Other ASD treatments are available for older children and adolescents through the age of 18 [17]. Children diagnosed with other developmental or mental disorders largely do not benefit from these services. Thus, the shared symptomology between disorders, coupled with heightened awareness and public de-stigmatization of ASD, and with the availability of ASD-specific services that are not accessible to children diagnosed with other conditions, may have created incentives for the over-diagnosis of ASD. Analogous reasoning could be argued for the similar overall responses provided by physicians for ADHD (Tables 2 and 3), since comparable incentives, including educational accommodations and access to cognitive enhancement drugs (stimulants), are also tied to receiving an ADHD diagnostic label [18].

The sharp increase in ASD diagnosis caused the Israeli Ministry of Health to change the criteria for receiving benefits and to implement new eligibility guidelines in 2007. The new guidelines require that ASD diagnosis previously given solely by a physician specialist be independently confirmed through a second evaluation by a qualified psychologist [19]. The DSM-5 criteria, introduced in late 2013, also aimed to increase the sensitivity and specificity of ASD diagnosis [20]. Nevertheless, despite these diagnostic changes, recent data suggest that after an initial plateauing [21], ASD incidence continued to increase [3, 4]. Therefore, it is paramount to continue efforts in precisely characterizing the signs of autism to increase the specificity and sensitivity of ASD evaluations. Continuous education of the medical team and peer learning could improve the quality of the evaluation process and enable a more detailed and accurate diagnosis.

Additionally, children with other mental and neurodevelopmental conditions, including ADHD, conduct disorder, and social anxiety, might need more treatments and individual help in school than children with high functioning ASD. Since it is not feasible to provide unlimited services to all children, we highly recommend basing any decisions concerning treatment and service eligibility not only on having a diagnostic label but also on the level of functioning. Moreover, children with uncertain ASD diagnoses should be eligible for intensive initial treatments (like those with unequivocal ASD) with frequent reevaluations before a definite diagnosis is made. This is in accordance with the new Diagnostic Classification of Mental Health and Developmental Disorders of Infancy and Early Childhood (D.C.): 0–5, which suggests a diagnosis of early atypical ASD for young children with ASD features without complete symptomatology [22]. This diagnosis is less stable than ASD but will allow young children with partial symptomatology to get proper interventions.

The lack of information from the physicians that did not answer the survey is a limitation of the study. In Israel, most children with ASD are diagnosed before the age of 6 in Child Development Centers, which could explain the higher survey response rate of child neurology and development specialists who more often work in these centers.

## Conclusions

Our survey of specialists in child development, neurology and psychiatry indicated that most physicians believed that ASD incidence is increasing, with some of this increase attributable to diagnoses given despite the clinical evaluation being inconclusive. Decision-makers are advised to adopt an approach that services should be gated by function regardless of diagnosis in determining eligibility for treatments and services. The medical community, for its part, must strive to improve diagnostic accuracy and for continuous review of diagnostic criteria.

## Data Availability

The datasets used and/or analysed during the current study are available from the corresponding author on reasonable request.

## Abbreviations

ASD: Autism spectrum disorder
CP: Cerebral palsy
ADHD: Attention deficit hyperactivity disorder
